# Confusion Worse Confounded

**Published:** 1891-10-10

**Authors:** 


### The Hospital

A Weekly Journal of

Science, Medicine, Nursing, and Philanthropy.

*For Hospitals, Asylums, Infirmaries, Dispensaries, Medical Practitioners, Students, Nurses, and the Charitable Public.*

Vol. XI.—No. 263.] [Oct. 10, 1891.

### CONFUSION WORSE CONFOUNDED.

In the multitude of counsellors there used to be safety; there is now confusion, blank and hopeless. On three succeeding pages of a daily newspaper last Friday, three representative men spoke their minds in an incidental kind of way upon things in general. Mr. Andrew Lang, the genial apostle of universal knowledge, gave a presidential address at the Folk-lore Congress; and he took occasion to remark that mankind as a whole would never be made scientific. Even men of science, like Ixion, he said, had embraced agreeable shadows and disembodied mediums.

Sir George Humphry, M.D., F.R.S., also spoke his mind. Sir George, too, was delivering a presidential address, to the students of St. Thomas's Medical School at the opening of their winter session. The learned medical knight evidently considers science as generally indispensable as Mr. Andrew Lang holds it to be generally impossible. Said Sir George, the most important change which had taken place in medical study during the last half century was an increase in accuracy; and it was by accuracy, that is, by the scientific method, alone that all good work could be done. Accurate work was good work, and inaccurate work was bad work. In fact, accuracy was only another word for attention and painstaking study. This utterance is surely most true, though it as certainly is not new. It is an oracle, if somewhat of a common-place one.

The third among the counsellors of last Friday's newspaper readers was Mr. Gladstone. He also delivered a presidential address, on the occasion of the jubilee of a Scotch public school. Mr. Gladstone undertook the defence of the clerical intellect; a chivalrous undertaking worthy of his intellectual history. It had been proposed, he said, that the cathedrals of England should be secularised, and should be placed in the hands of lay persons, and that they should no longer remain the clerical establishments which they now were. The ground of this proposal was that the clergy were falling altogether behind in the intellectual race, that, to use a familiar phrase, "they are nowhere." He believed that to be most inaccurate, most untrue, and an intentional and unjust aspersion. They might judge of the character of a body in part by the names of those who die in its ranks. He named five names of men who had died in the ranks of the British clergy within the last two years. One was Bishop Lightfoot, one was Dr. Liddon, one was Dean Cqurch, one was Archbishop Magee, and one was Mr. Aubrey Moore, a man to whom all persons who were familiar with the affairs of the University of Oxford looked with the greatest admiration and interest, because they knew the powerful contribution he made to the thinking power of the clergy of the country. The body was an illustrious body from whose ranks within less than two years five such men were to be numbered. Mr. Gladstone protested most emphatically against the frequently expressed opinion that if young men wanted to be men of intellect they must avoid the clerical profession. Let them rely upon it that the objects of that profession were the highest objects that any man could propose to himself.

It was said of a certain lover that when he finally found out, after many suspicions, that his angel was a downright flirt, he "did not know whether to laugh or to cry." And really when one thinks of the gay and airy Mr. Andrew Lang at one end of a see-saw, and the tremendously serious Mr. Gladstone at the other end, with the "accurate" Sir George Humphry balancing them in the middle, one does not know whether to believe on the one hand, that "All the world's a stage and all the men and women merely players"; or on the other that "Life is real, life is earnest, and the grave is not its goal." Even Mr. Gladstone himself, and Sir George Humphry, would admit that a young man of enquiring mind, coming casually across three such different views of life, from three such competent persons, on three successive pages of the same newspaper, would be entitled to hold his breath with a considerable degree of amazement.

What is a man to believe? The question is as practically important as that of one's annual income. A man is strong and capable in proportion as he has definite convictions. "He who would do faithfully, says Carlyle, "must needs believe firmly." There is no fact in all the range of science more certain than this. The absence of belief is the absence of backbone. The want of conviction is the want both of motive power and of staying power. A confused mind leads to confused purposes, or to no purposes at all: and the man who sits on the fence for ever can neither plough, nor sow, nor reap, nor take part in the battle.

But even Mr. Andrew Lang, butterfly as he sometimes seems to be, is really after all a bee. If he be observed long enough it is seen that he is gathering honey, as Dr. Watts puts it, "from every opening flower." The honey that he gathers is that kind of full and comparative knowledge which brings tolerance. A man like Mr. Andrew Lang could never be Pope of Rome, or even a cardinal. He is a standing protest against one of the most tremendous and dangerous of all errors, the error of human infallibility. Although he thinks the world can never be made scientific, he is himself an embodiment of the purest science. So far as in him lies he seeks knowledge pure and simple: he speaks that which he knows, and testifies what he has seen. That is to say, he explains the methods of his research; he gives the results as he finds them; and he claims no more for them than what they are justly worth.

Professor Humphry's gospel of accuracy is only the contraction into a formula of Mr. Lang's praise of full knowledge on every subject open to the explorer's eye. Of course we must be accurate. The man who does not strive to be accurate is an inconsequent blockhead; the man who is wilfully inaccurate is dishonest. Medical men cannot claim the merit of having discovered the method or the value of accuracy. The old-world architects and builders have left us enduring evidences of the painful care with which they planned and executed their work. The Dutch and other great painters left nothing undone to make every detail of their work finished and complete. Even the schoolmen spent hours, days, weeks, nay, years in formulating single propositions, in order that those propositions might be so exact and nice that no possible objection could be urged against them. The very fact that they were charged with discussing the absurd question, how many angels could dance on the point of a needle, shows how widespread and notorious was their reputation for painstaking minuteness. The remains of Greek and Egyptian art constitute standing monuments of persistent, painstaking, and thoughtful labour, devoted to great and noble ends.

It is hardly open to question that the mind of Mr. Gladstone is more comprehensive than that of Sir George Humphry, and more forcible and weighty than that of Mr. Andrew Lang. The greater includes the less. The affirmation of an all comprehensive intelligence, and of eternal justice and truth, includes the tolerance of all kinds of subordinate intelligence and its work; includes accuracy and thoroughness as necessary details. The clergy are set apart to a truly scientific work; the work of selecting, discovering, and teaching the principles and details of morals. There can be no more truly scientific work than this, no work that is greater or more essential to the happiness and well-being of man. Mr. Gladstone selected names which were inferior to none in true and comprehensive science. Religion has an undying part to play in the world. If all its teachers were as essentially scientific as Westcott, Lightfoot, Church, Magee, and Aubrey Moore, no weapons of scepticism could for a moment prevail against them. Whatever difference of opinion there may be about theological details, the philosophic mind can never fail to hold firmly by the conviction that mind, purpose, and justice are at the foundation of all that exists.

